# Supplementary figures and images for: Genomic Analysis of Romanian Lycium Genotypes: Exploring BODYGUARD Genes for Stress Resistance Breeding
Source: Int J Mol Sci. 2024 Feb 9;25(4):2130. doi: 10.3390/ijms25042130 (PMC10889844; doi:10.3390/ijms25042130)

Chromosome

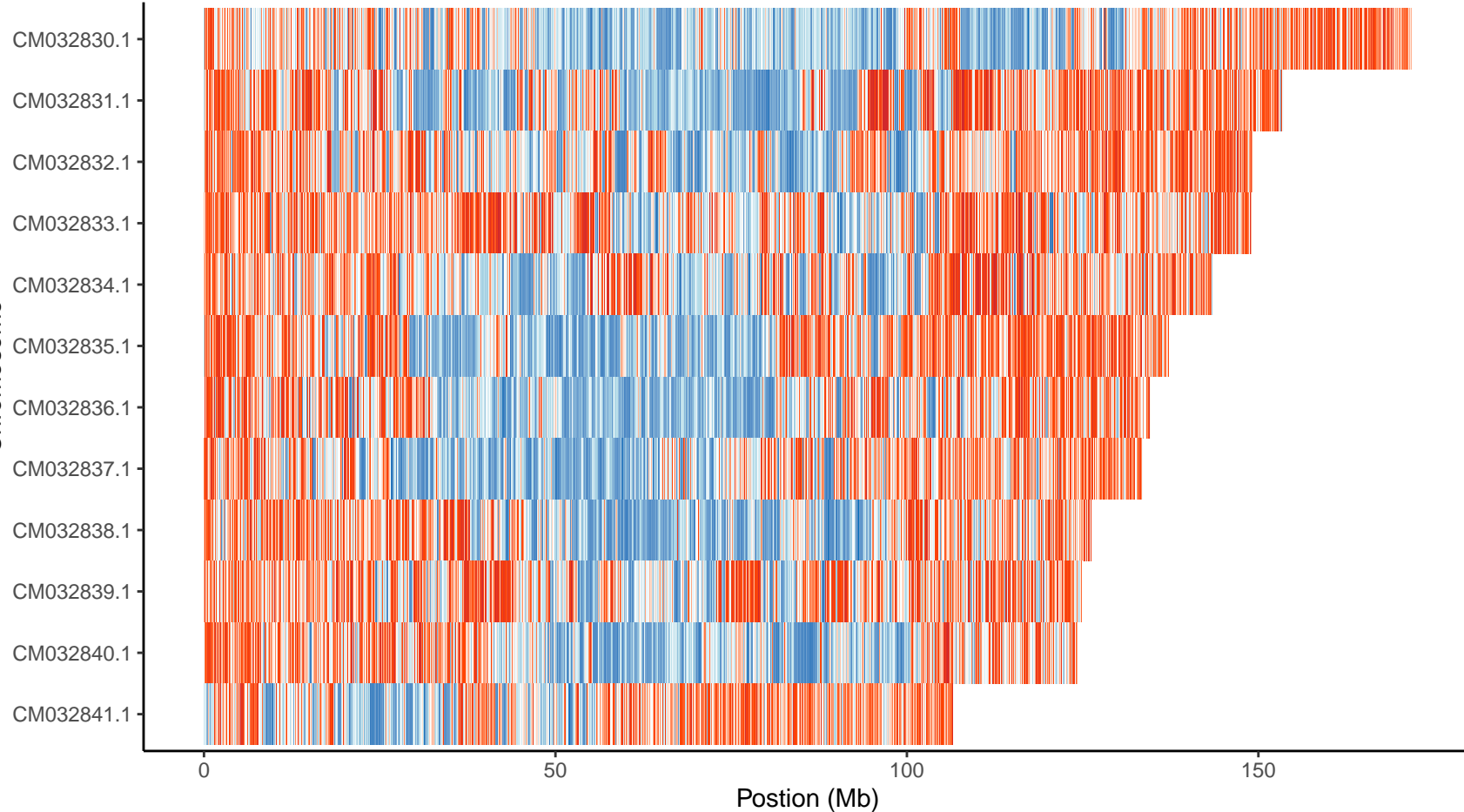

Chromosome

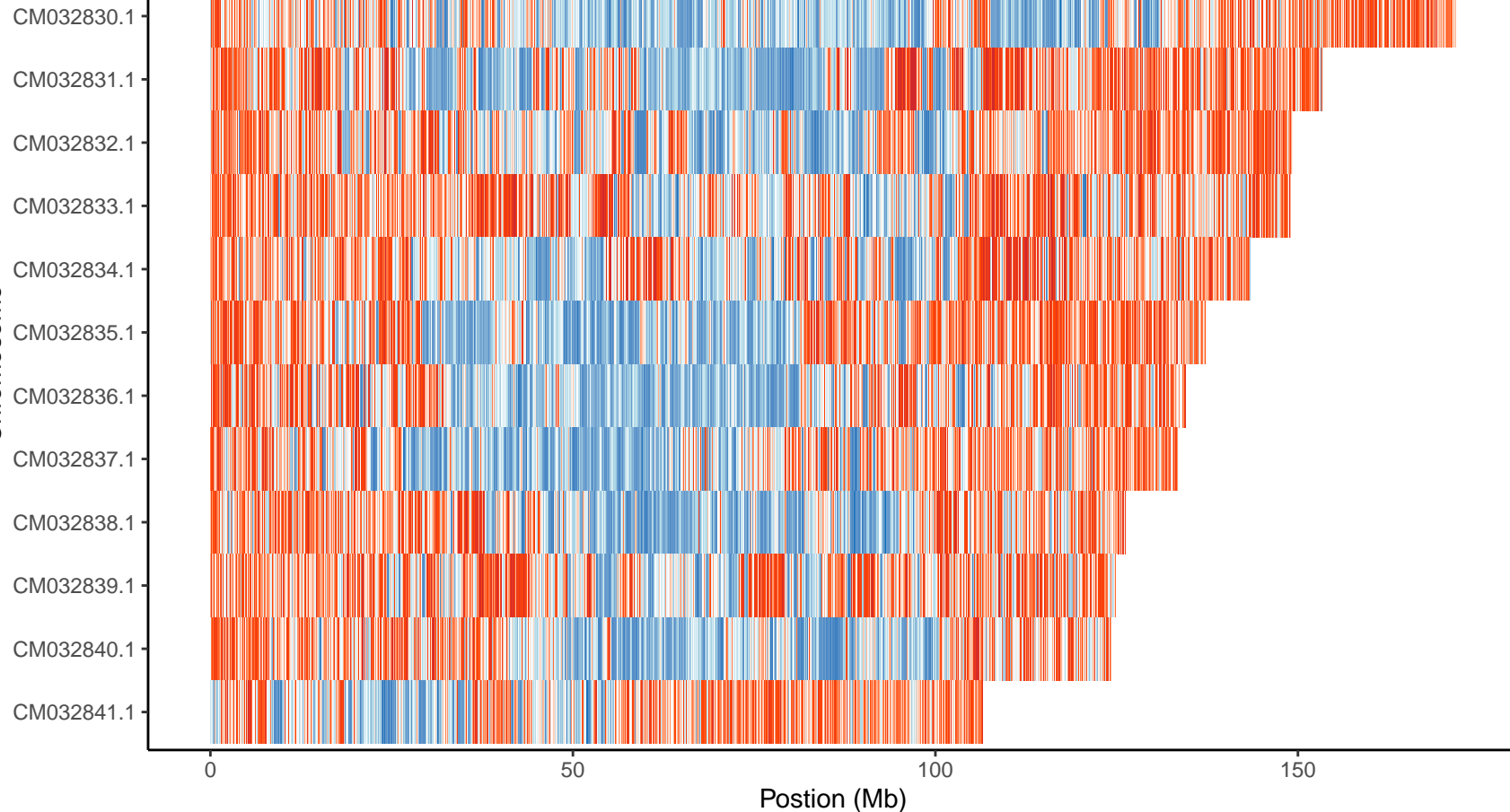

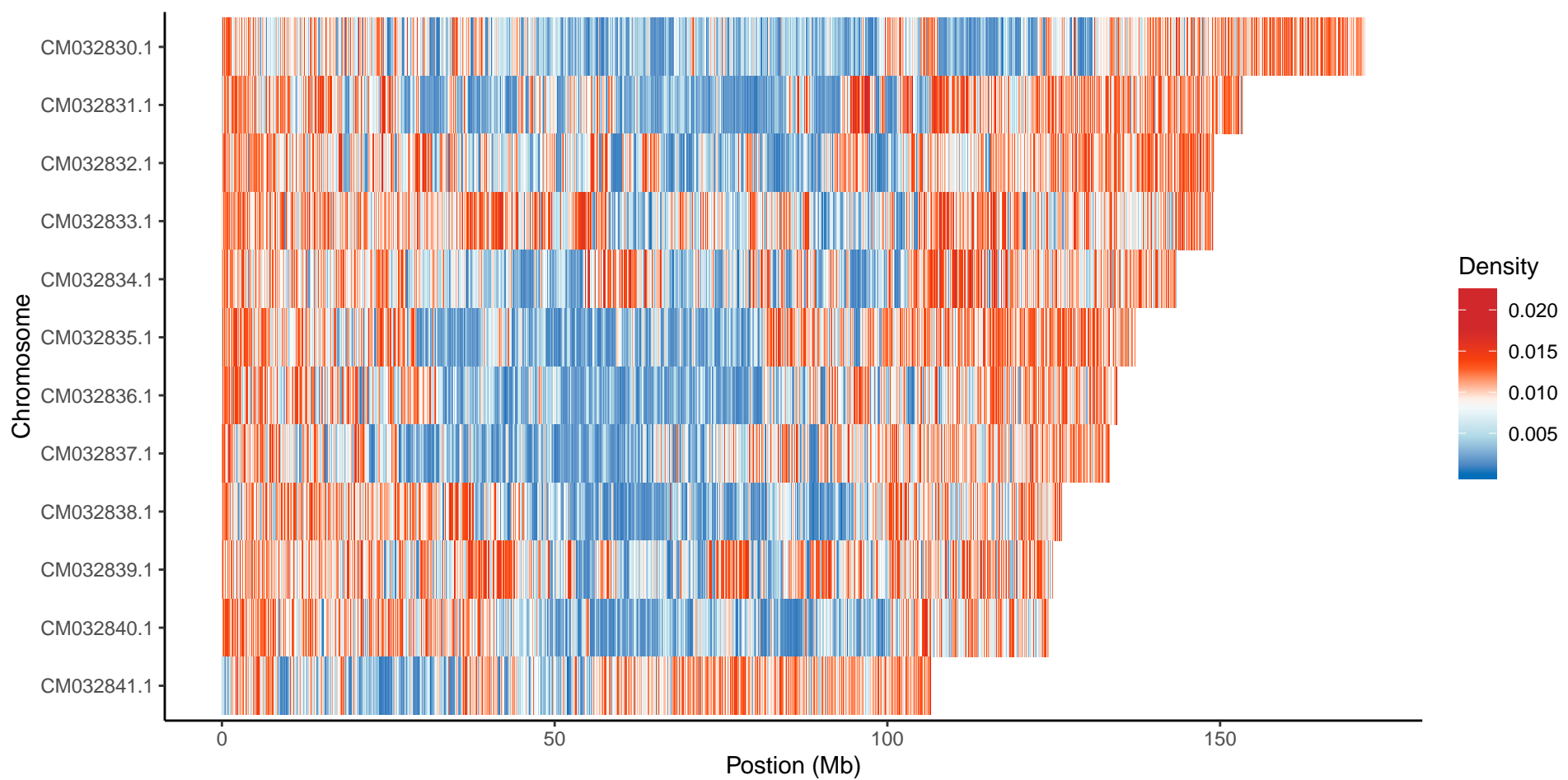

Chromosome

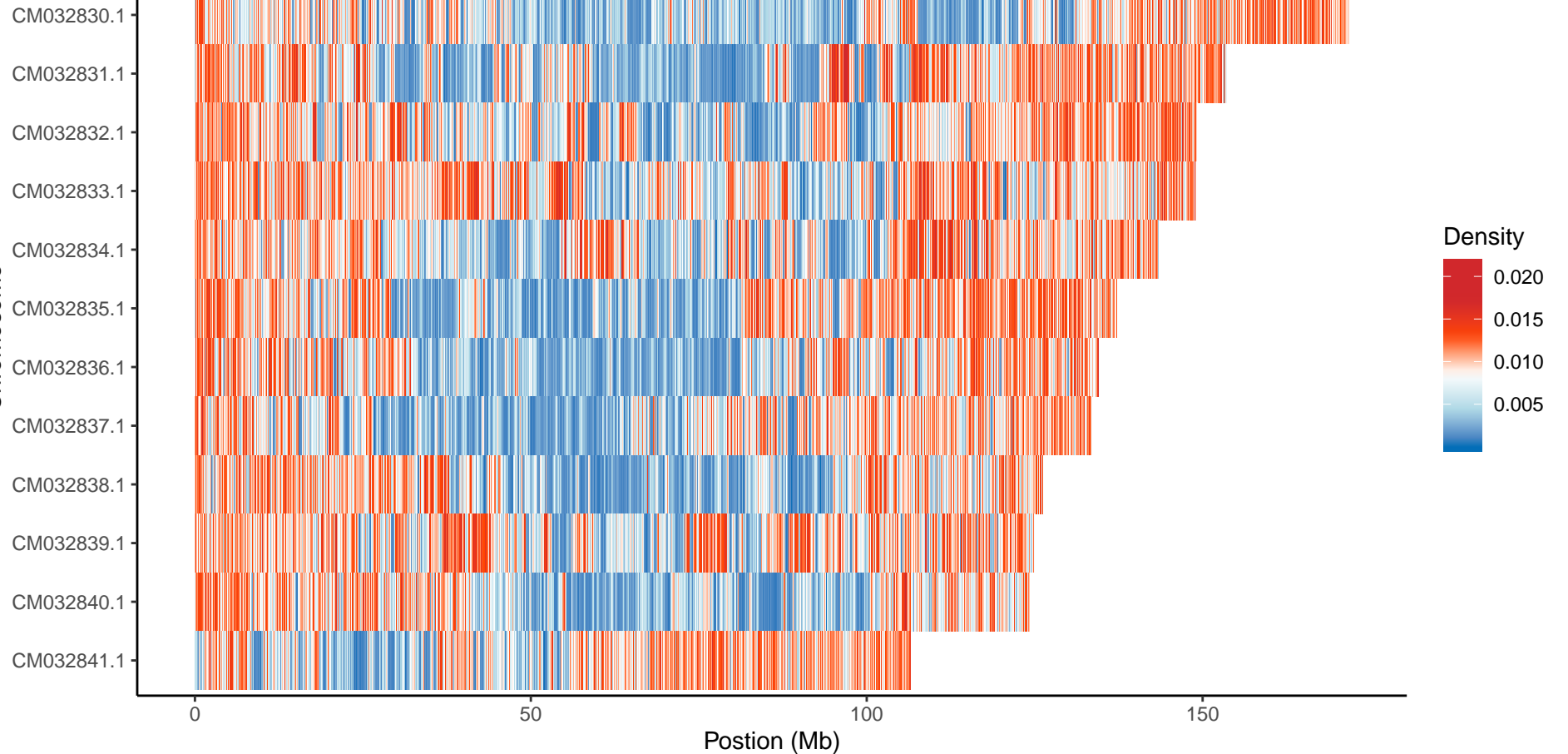

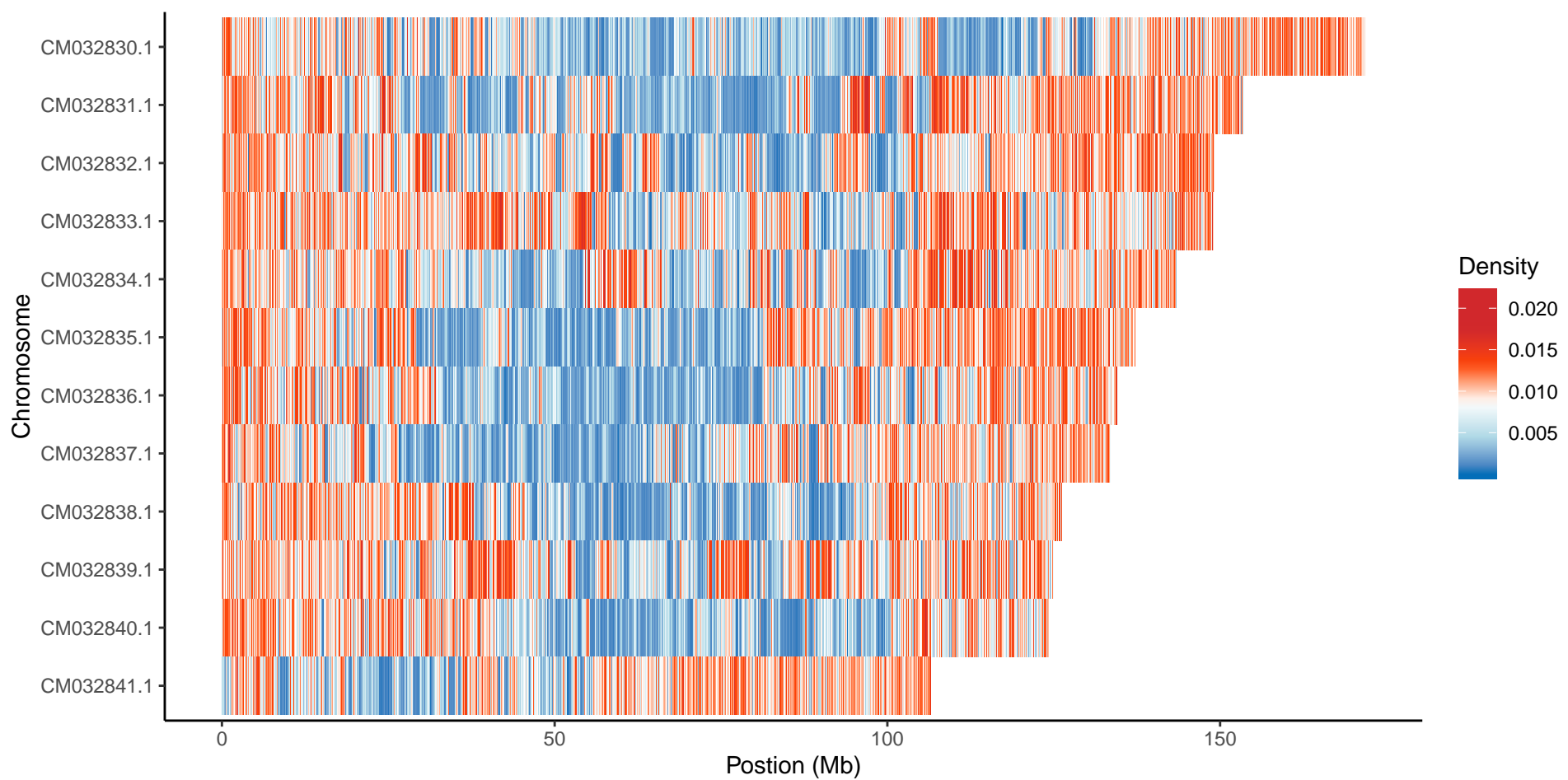

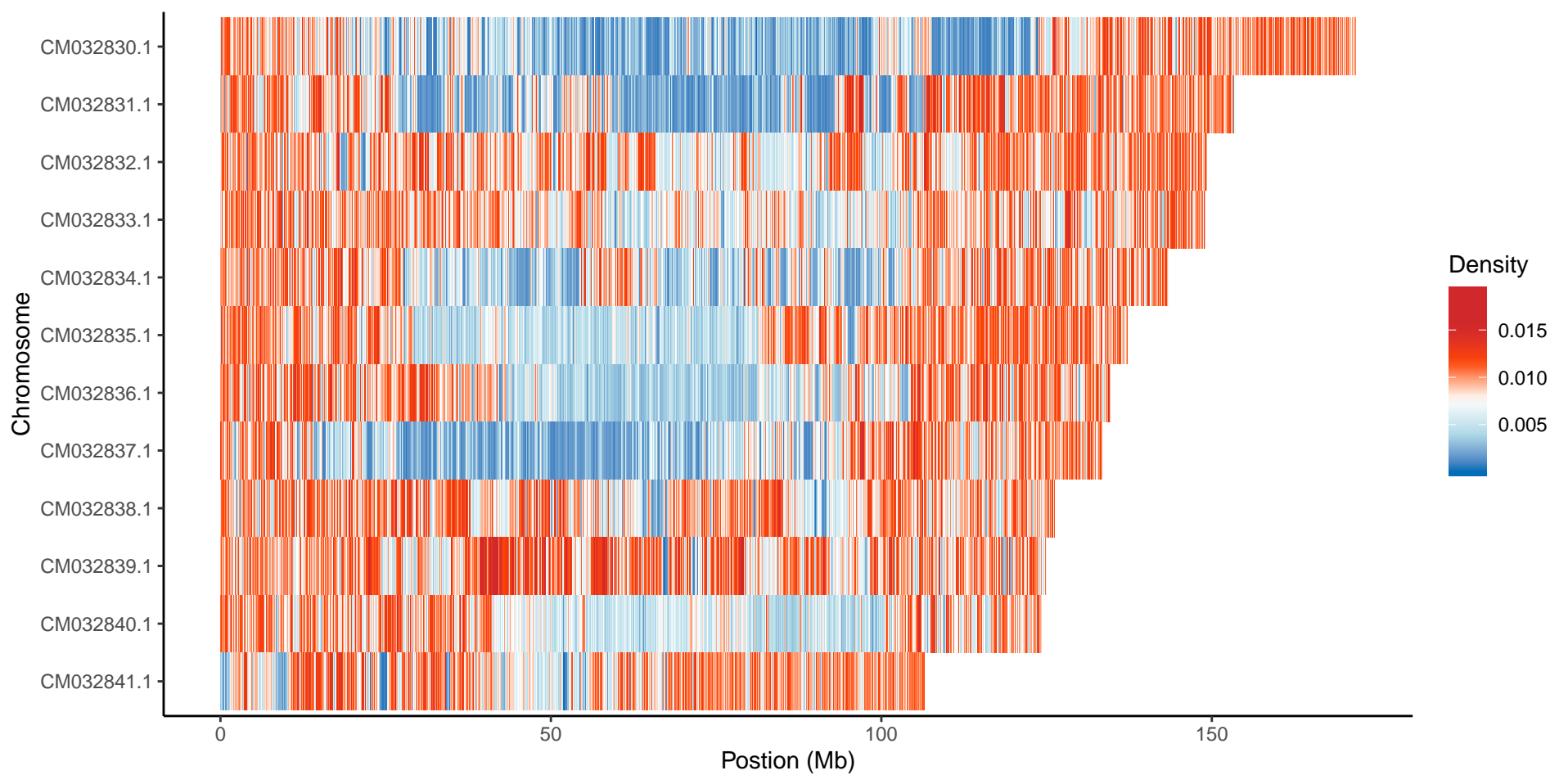

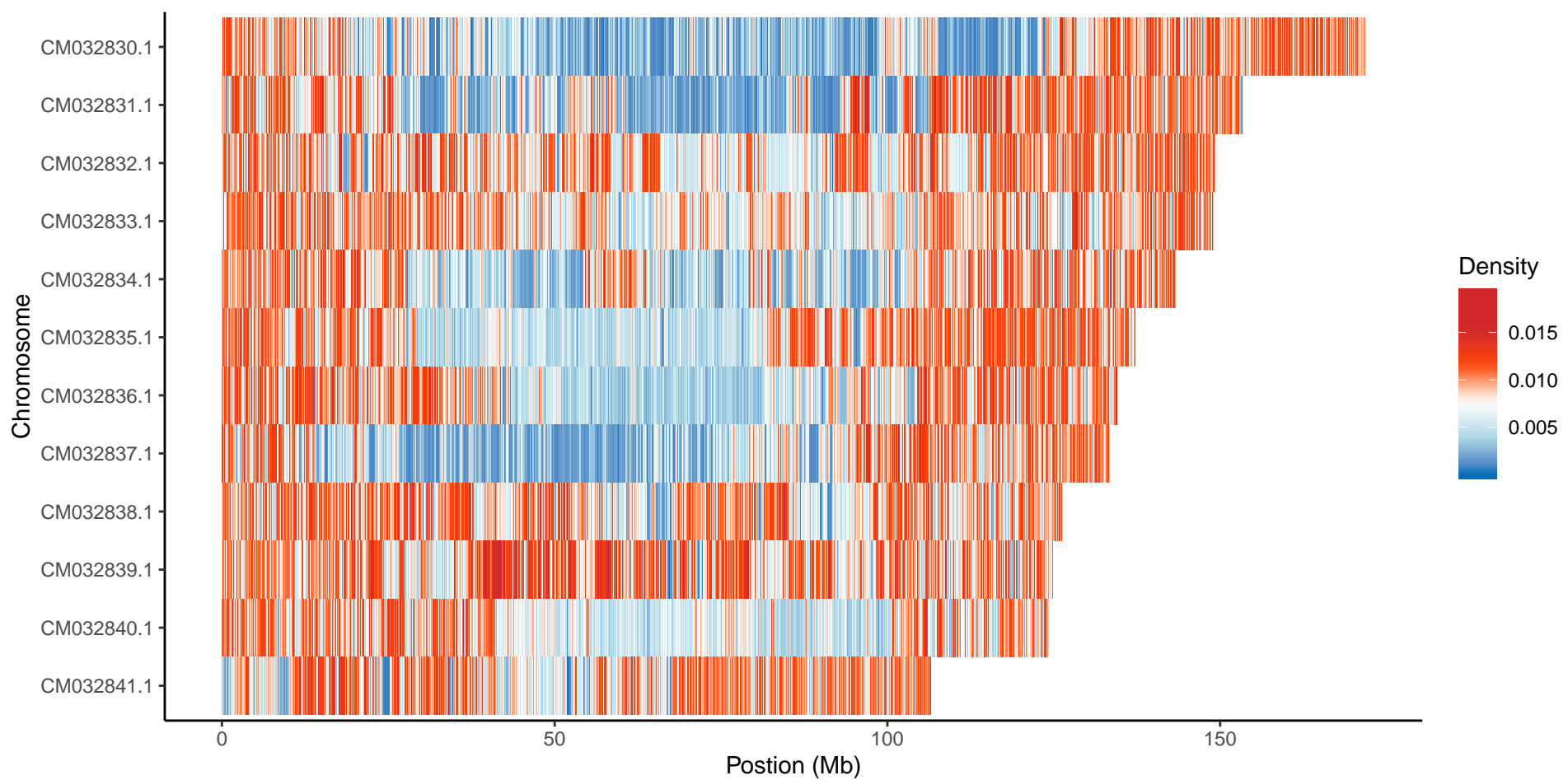

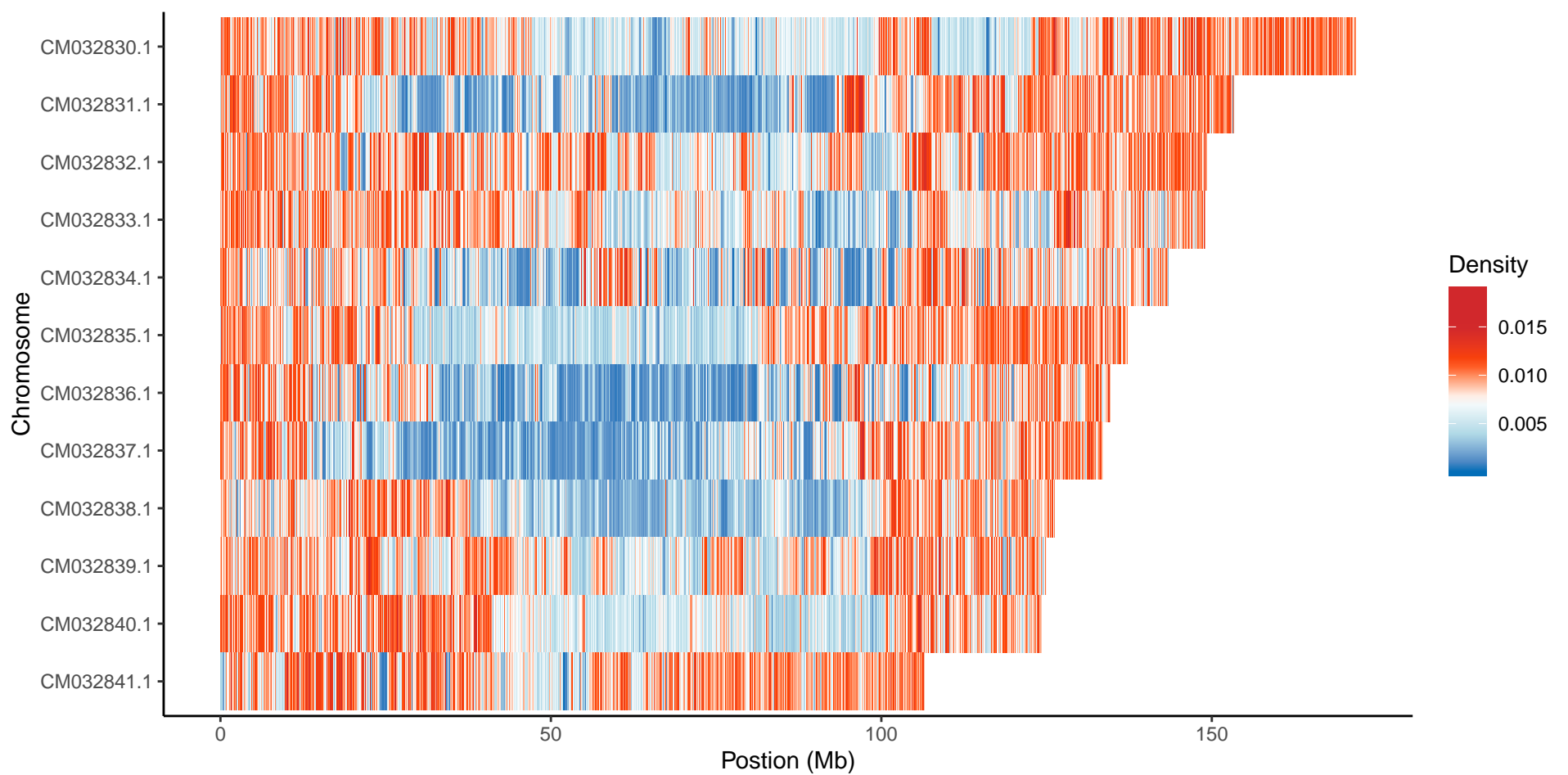

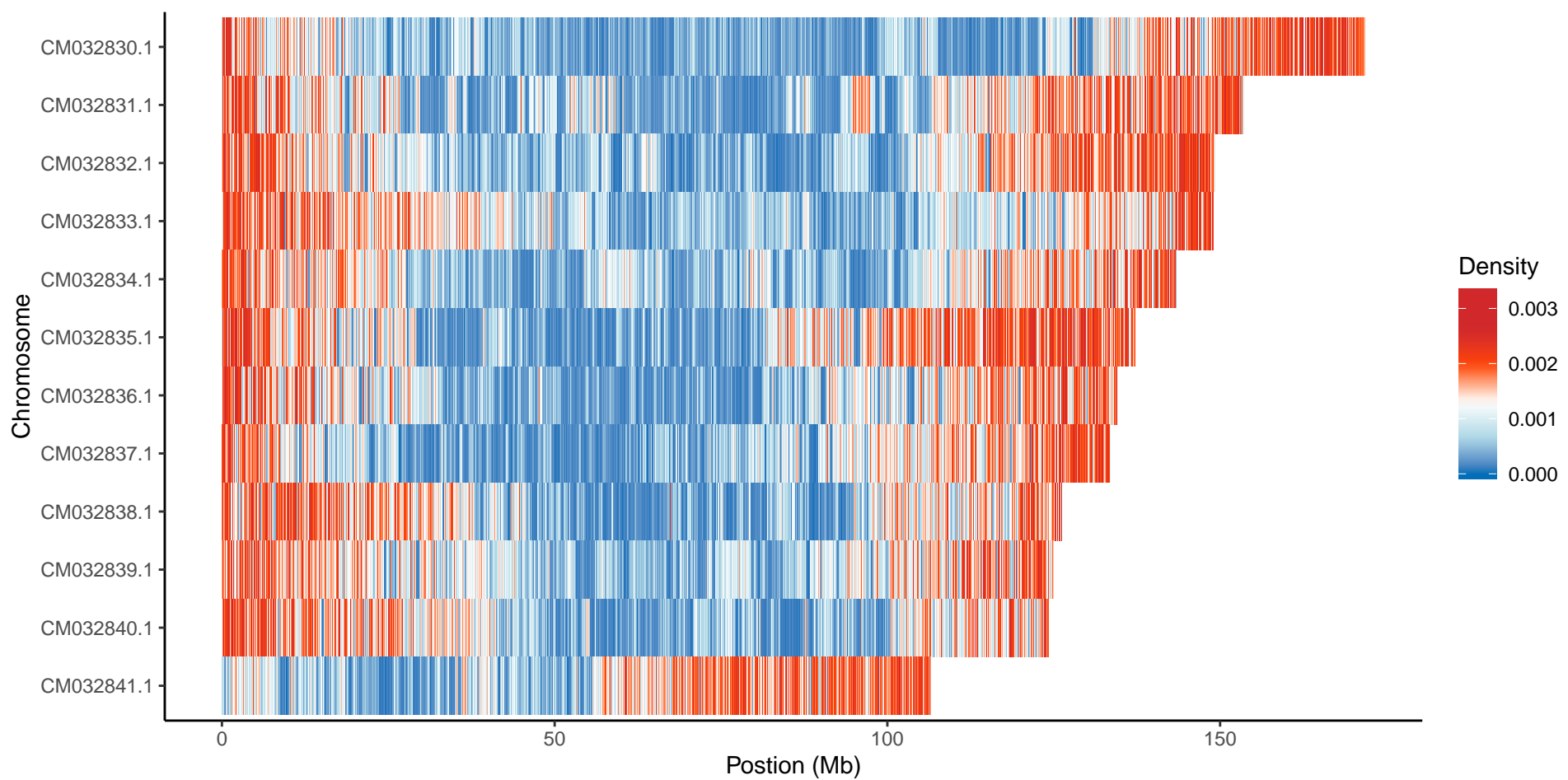

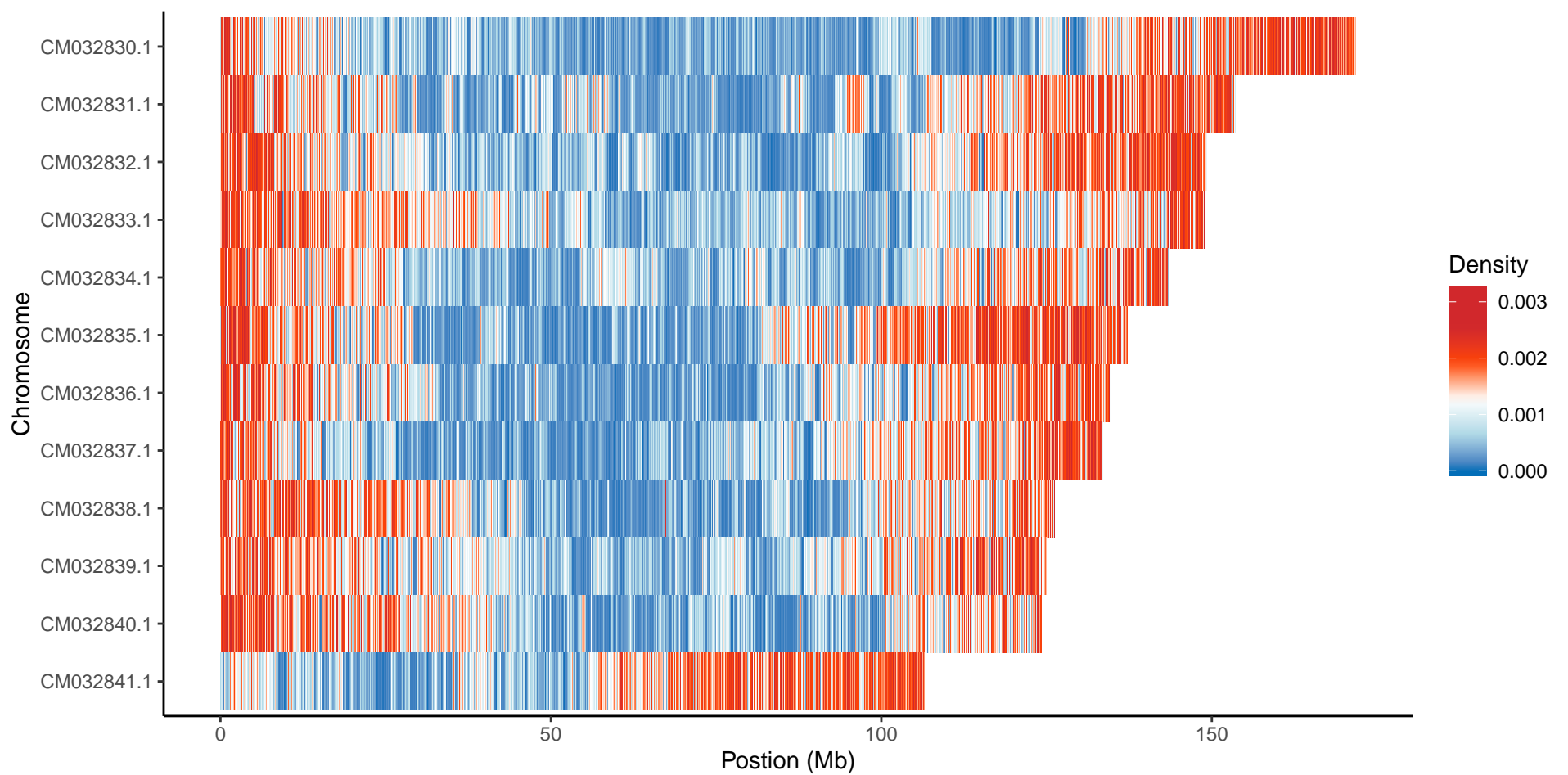

Chromosome

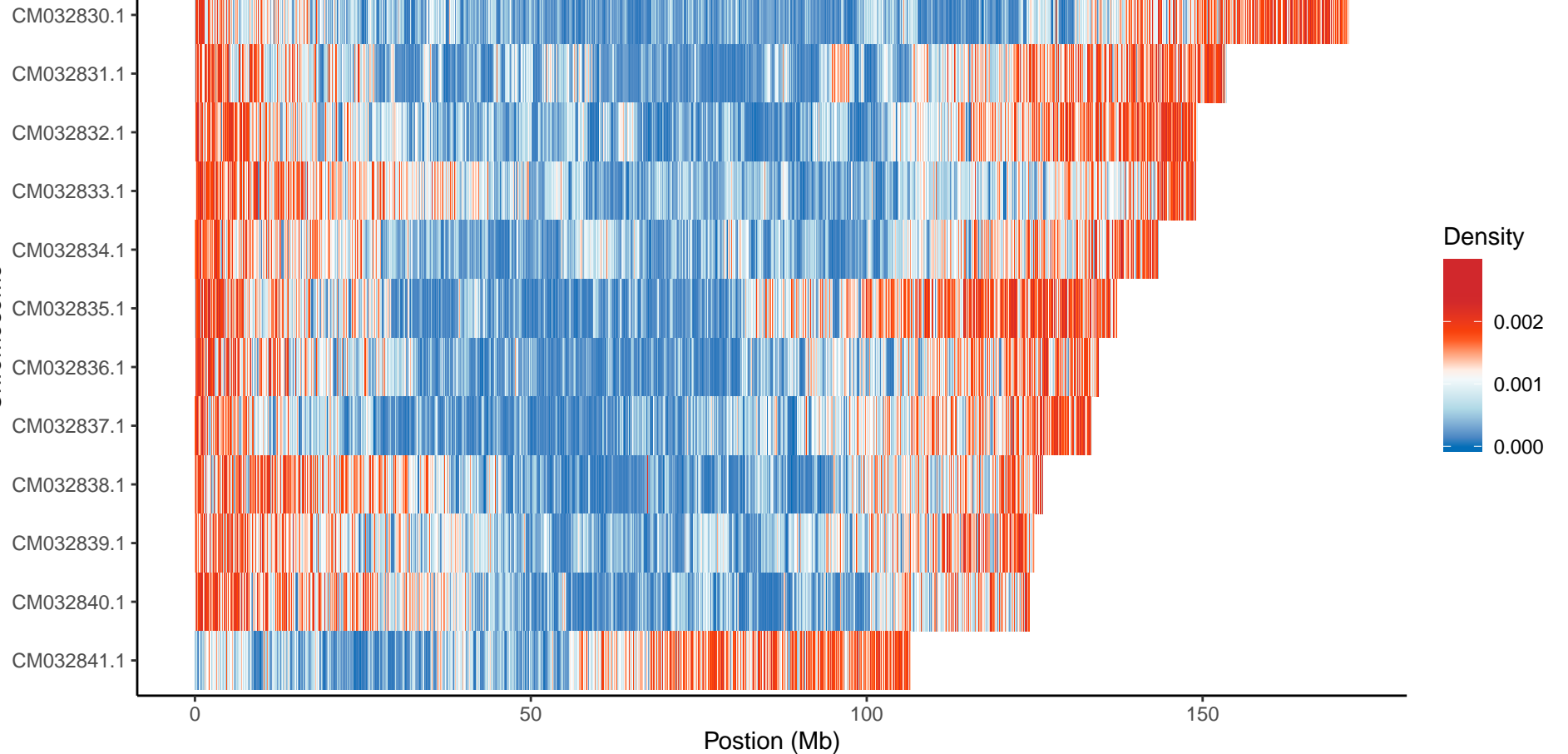

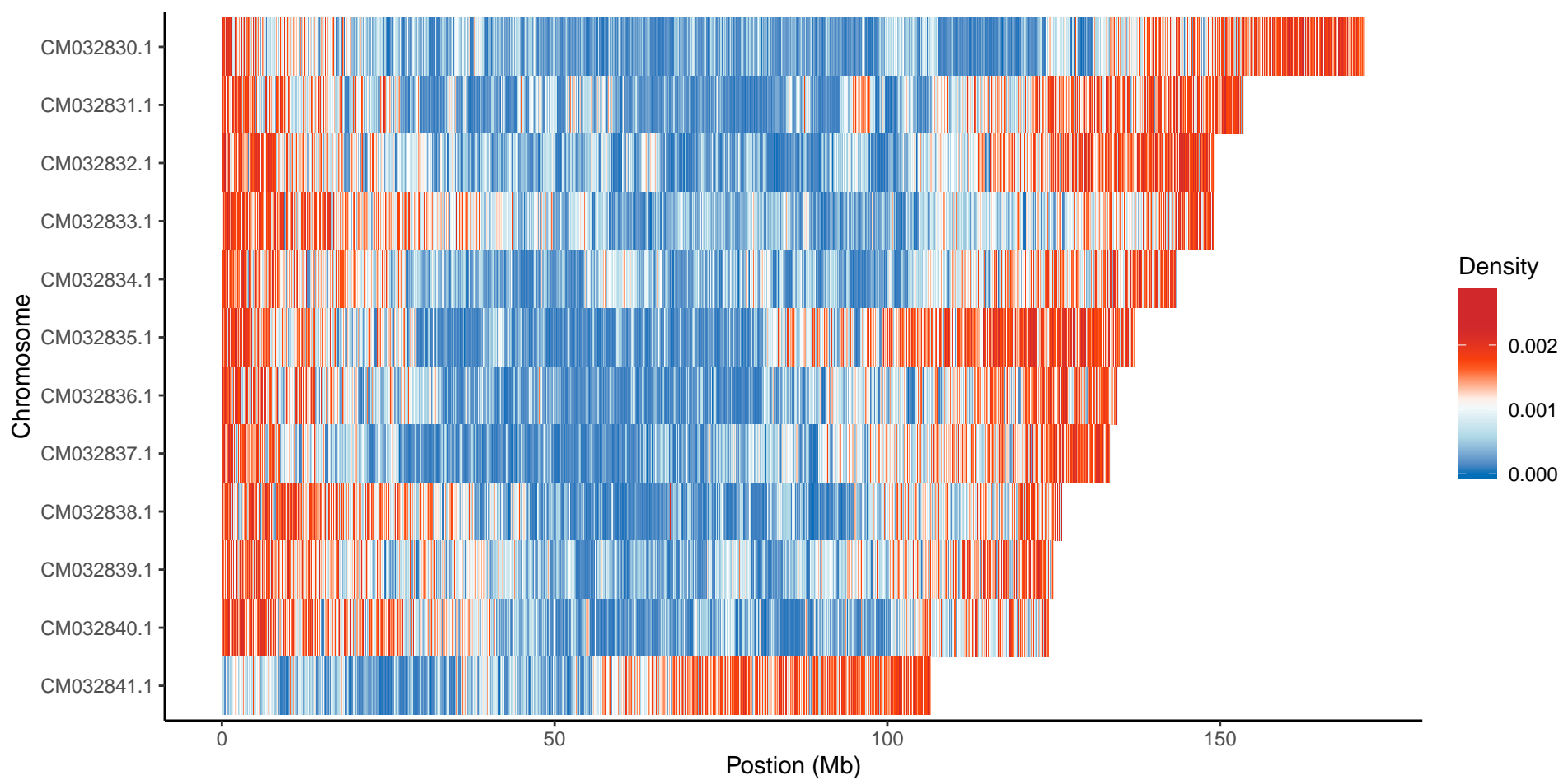

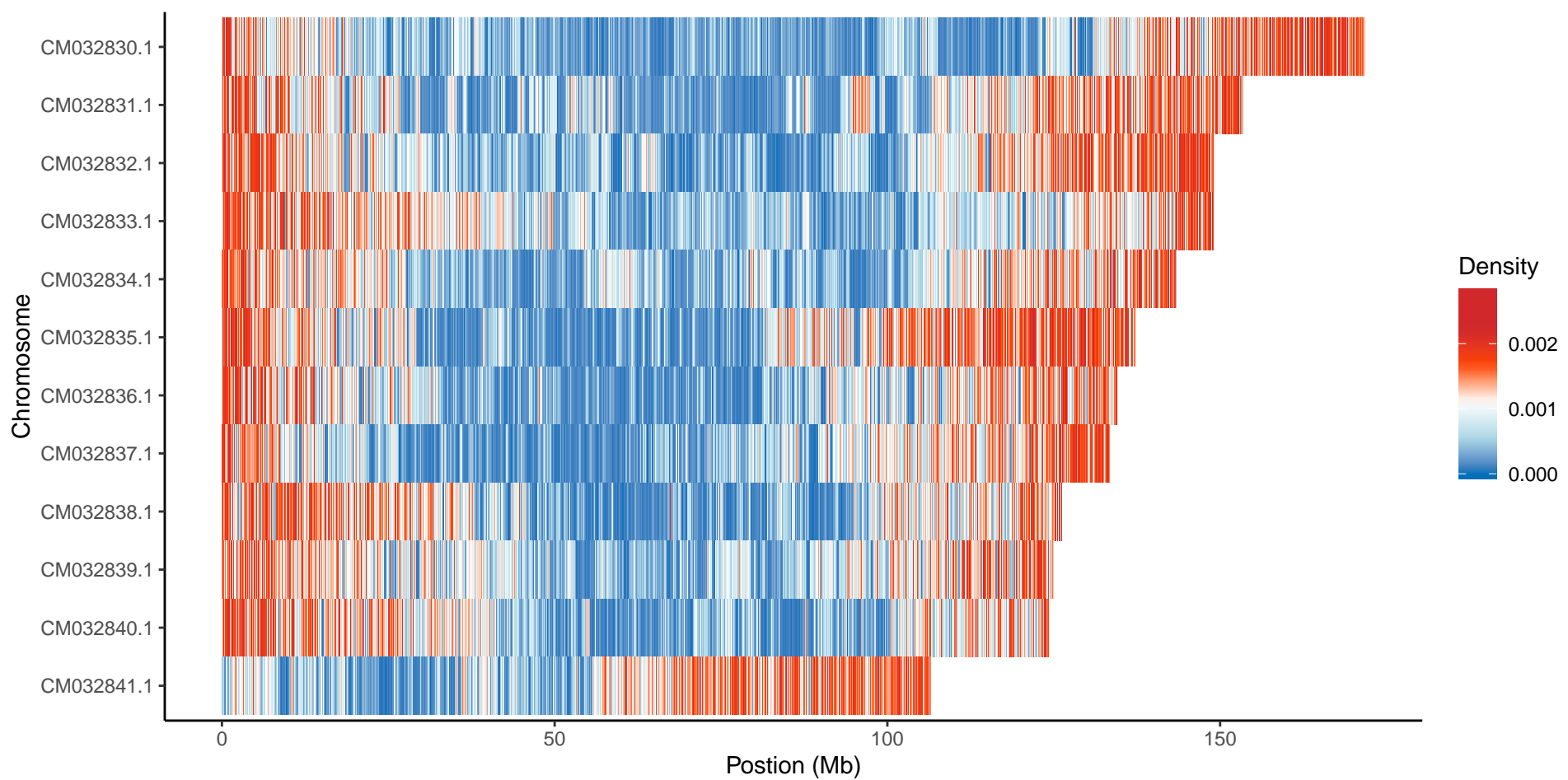

Chromosome

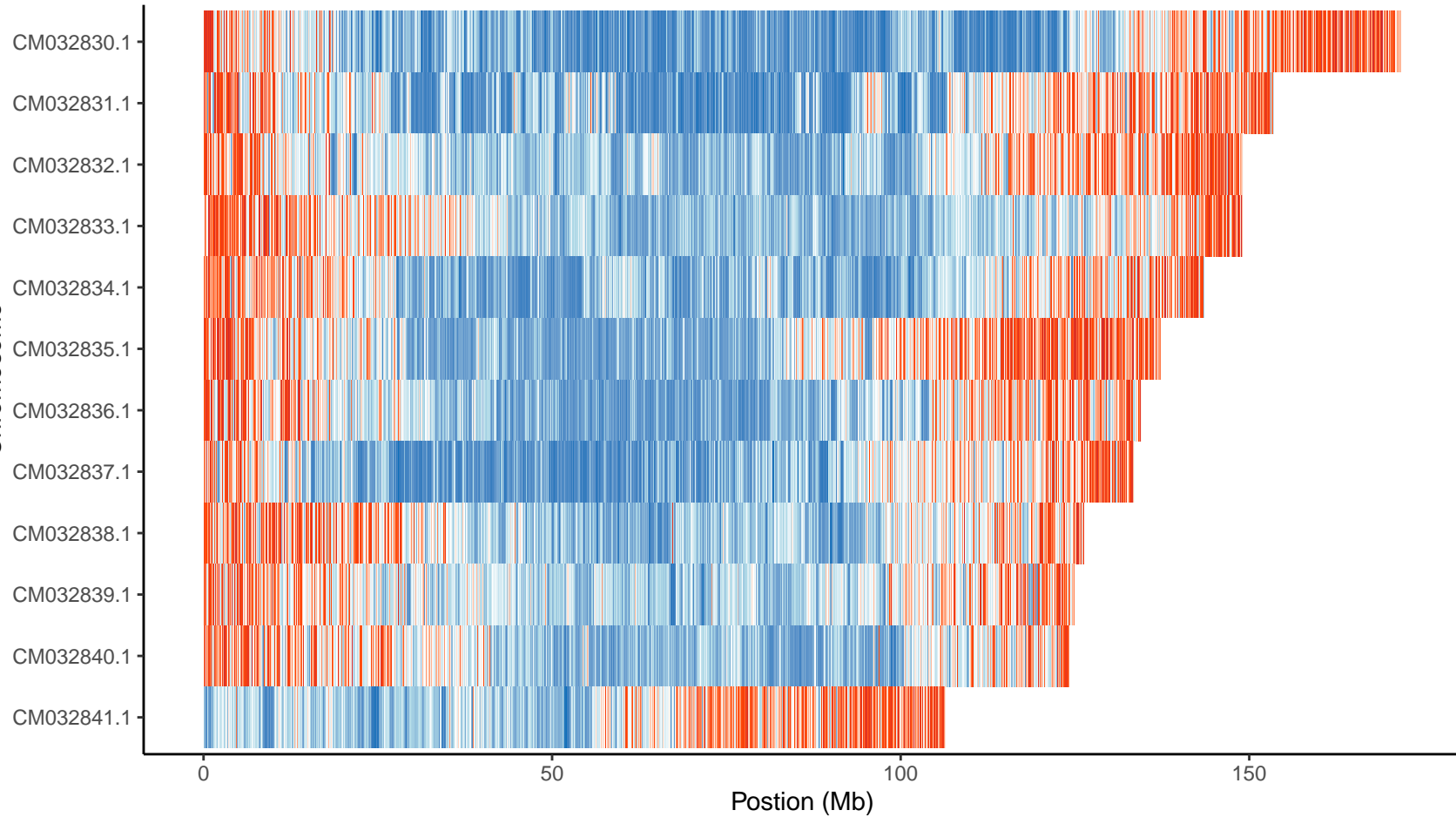

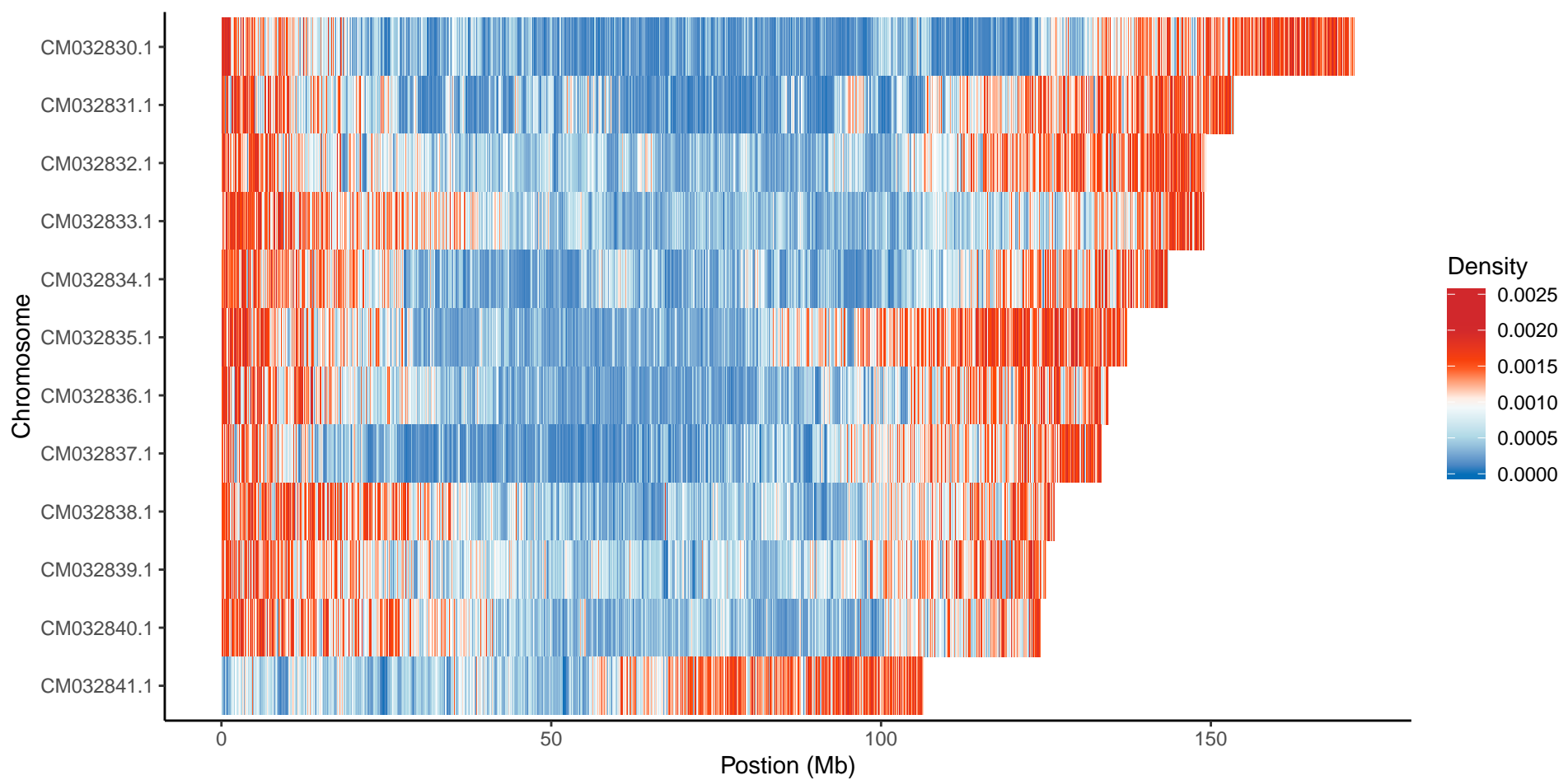

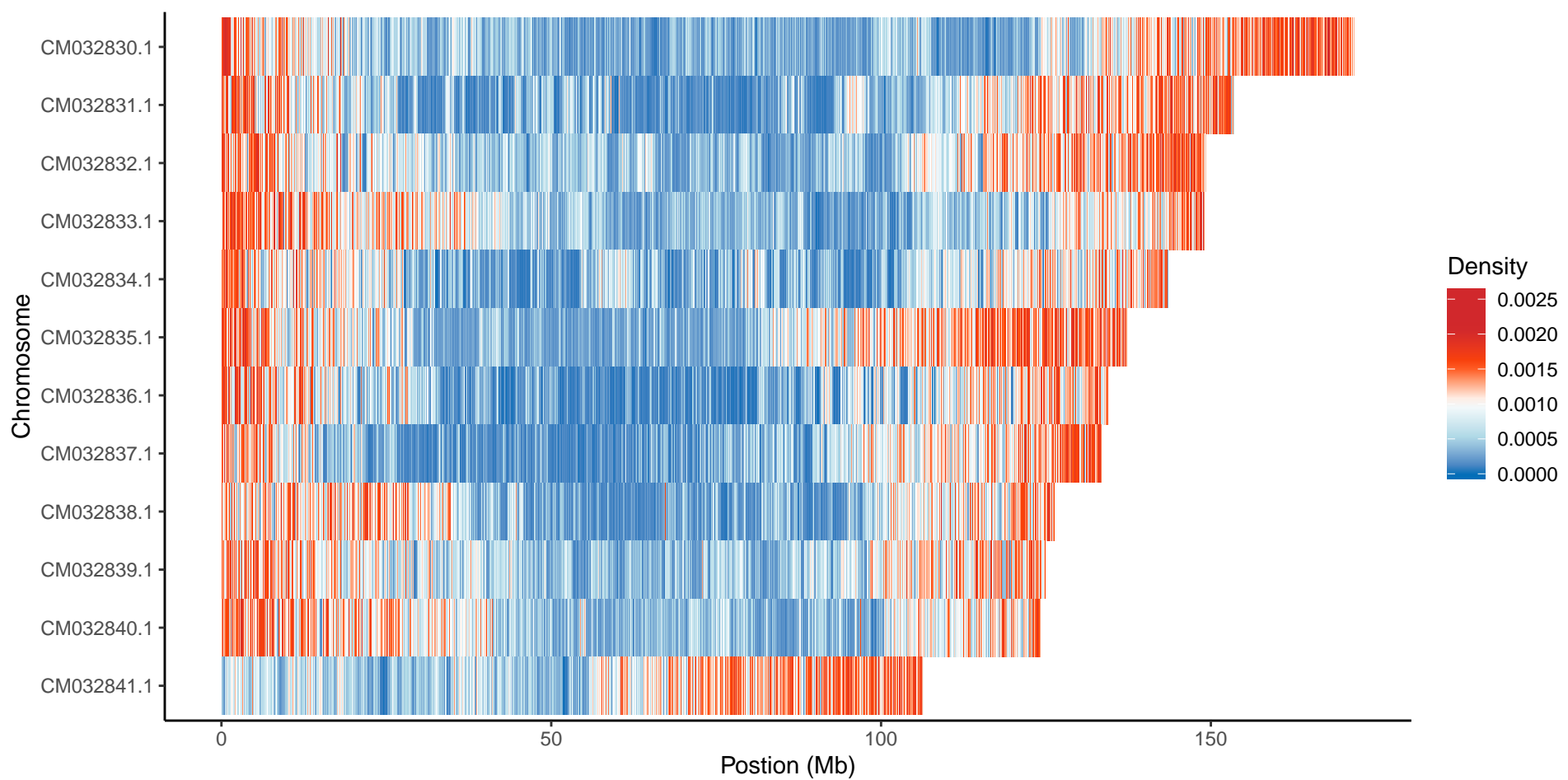

Supplement: Supplementary file 1 [file ijms-25-02130-s001.zip › Supplementary file S2_Ciceoiet al._06.VarDetect_Visualization.pdf]
